# Healthcare Providers’ Experience in Implementing the Adolescent and Youth-Friendly Services at Public Health Facilities in KwaZulu-Natal: A Qualitative Study

**DOI:** 10.3390/healthcare13162033

**Published:** 2025-08-18

**Authors:** Patience Primrose Khuzwayo, Sipho Wellington Mkhize

**Affiliations:** College of Health Sciences, Discipline of Nursing, Howard Campus, University of KwaZulu-Natal, 238 Mazisi Kunene Road, Glenwood, Durban 4041, KwaZulu-Natal, South Africa

**Keywords:** adolescent health, youth-friendly service, implementation, experience, healthcare provider

## Abstract

**Background/Objectives**: The adolescent and youth phase is characterized by rapid physical, cognitive, and psychosocial development during which adolescents encounter numerous challenges. These challenges include experiences of sexual violence, sexually transmitted diseases, mental health issues, poverty, lack of education, social discrimination, and high fertility rates. The Adolescent and Youth-Friendly Services (AYFS) program in South Africa aims to enhance young people’s access to sexual and reproductive health services (SRHS). This study explores the healthcare providers’ (HCPs’) experiences in implementing the AYFS within public health facilities in eThekwini, KwaZulu-Natal. **Methods**: This exploratory, descriptive qualitative study employed individual in-depth interviews to gather data from eight HCPs. The target population consisted of HCPs working in the public health facilities that offer AYFS. A purposive sampling technique was utilized to select HCPs who met the inclusion criteria. Thematic analysis was conducted following the steps outlined by Braun and Clarke. **Results**: The participants consisted of black females and one male, aged 34 to 50, with 1 to 14 years of experience. The four main themes emerged from the study: appropriate service provision, HCPs’ competency, accessibility of AYFS, and adherence to the principles of beneficence and non-maleficence. Overall, HCPs reported a positive experience in implementing AYFS. **Conclusions**: The findings indicated that AYFS was delivered as a comprehensive package addressing the needs of adolescents; however, there is a pressing need to enhance demand-creation initiatives in schools and communities to raise awareness and promote service utilization among this vulnerable population. The implications of these findings are to ensure thorough implementation and utilization of AYFS in the country.

## 1. Introduction

The global population comprises approximately 1.3 billion adolescents [1], with a significant concentration in sub-Saharan Africa; specifically, South Africa is home to 30% of adolescents aged between 10 and 24 years [2]. In East and Southern Africa, an estimated 32% of the population falls within the age range of 10 to 24 [3]. The adolescent phase, as defined by the World Health Organization, encompasses individuals aged 10 to 19 and is characterized by rapid physical, cognitive, and psychosocial development [4]. Adolescent boys, and particularly girls, are at high risk of exposure to sexual violence (37.9%,) sexually transmitted infections (35.3%), elevated fertility rates (48.7%), mental health concerns (17%), social discrimination (59.3), poverty (62.1%), and limited educational opportunities (52%) [5,6,7,8,9]. It was reported that one in five girls becomes pregnant in South Africa before the age of 20, and within a period of one year, over 90,000 girls aged 10–19 gave birth [10]. This was linked to poverty, community norms, and lack of access to contraceptives [10]. The lifetime and past-year prevalence of sexual violence was 37.9% and 25.3% among adolescent girls and young women in South Africa [6]. Young people in KwaZulu-Natal, in addition to the above-mentioned challenges, also experience single-parent or child-headed homes and abandonment [11].

Adolescent and Youth-Friendly Services and Sexual and Reproductive Health and Rights (SRHR) were established to address these challenges. AYFS is an evidence-based health intervention aimed at alleviating sexual and reproductive health issues, as well as other challenges faced by adolescents and young adults [12]. The World Health Organization defines AYFS as services that are accessible, acceptable, equitable, appropriate, and effective [13]. AYFS are characterized by their ability to attract adolescents, provide a welcoming environment, meet specific service delivery needs, and maintain engagement [14]. Additional components of AYFS include creating a comfortable atmosphere for adolescents and fostering trusting relationships with healthcare providers [15]. The AYFS framework encompasses a variety of services, including SRH education, contraceptive counseling, human immunodeficiency virus (HIV) counseling and testing, and screening and treatment for sexually transmitted infections (STIs). Training HCPs in adolescent health is critical for delivering youth-friendly care [16]. Adolescents seek to be respected, heard, and free from judgment [17]. Consequently, it is essential for healthcare providers to possess the skills necessary to effectively address the needs of adolescents [18]. In this study, HCPs encompass nurses who render AYFS in the facilities.

In South Africa, the initiative targeting adolescents was initiated by Love Life, a non-governmental organization, in collaboration with the government. Between 2000 and 2005, the National Adolescent Friendly Clinic Initiative (NAFCI) was established, followed by Youth Friendly Services (YFS) from 2006 to 2011, culminating in the current AYFS model. YFS, in this context, are services designed to meet the unique needs of young people, ensuring they can access quality healthcare and information in a welcoming, respectful, and confidential environment. In the early 2000s, the focus was on HIV prevention and SRH services. The AYFS model includes an accreditation framework and is guided by standards to enhance adolescents’ access to quality healthcare services. The South African government has implemented several initiatives to enhance quality healthcare services for adolescents, including the Integrated School Health Policy (2012), the Adolescent and Youth Health Policy (2017–2022), the Child and Adolescent Mental Health (CAMH) policy guidelines (2003), and the Adolescent Sexual and Reproductive Health and Rights (ASRHR) Framework [19,20,21]. This program was implemented in community healthcare centers and comprehensive primary healthcare facilities. The AYFS program aims to improve the quality and accessibility of public health services for adolescents and youth [22].

The National Adolescent and Youth Health Policy 2017 [19] delineate AYFS interventions, which encompass the provision of a comprehensive service package for adolescents, training for HCPs, enhancement of digital education and support channels, implementation of integrated service delivery models, strengthening of referral systems where necessary, and ensuring accessible linked services for adolescents and youth. The AYFS targets individuals aged 10 to 24 years, categorized as adolescents [23]. This demographic encompasses both adolescents and youth, as specified in the South African Adolescent and Youth Health Policy 2017 [19]. Adolescents are entitled to SRHR, which grants them access to contraceptives, HIV testing, medical treatment, and prescribed medications at the age of 12, as well as the right to termination of pregnancy at any age and male medical circumcision at 16 years [24].

Kenyan literature reveals that insufficient training of healthcare providers, ineffective policy implementation, and low utilization of AYFS substantially impede the delivery of AYFS in public health facilities [25], while a Nigerian study revealed a negative attitude and less focus on the mental health of youths [26]. A study conducted in Limpopo province revealed the need for training of HCPs, allocation of resources, as well as a collaborative approach in the implementation of AYFS [27,28]. According to a study conducted in two South African provinces, neither sub-district’s facilities satisfied the minimal standards required to be recognized as the AYFS [29]. Despite the introduction of AYFS to address the needs of this age group, its implementation within the province remains inadequately documented [27]. According to a KwaZulu-Natal study, the main obstacles to the adoption of AYFS were inadequate infrastructure, a lack of employees, unfavorable staff attitudes, inadequate privacy, and a lack of communication [30]. The observed gaps pertained to insufficient monitoring and systemic issues, including adolescent health literacy and HCP training [28,31]. Consequently, there is limited evidence regarding HCPs’ experiences in executing AYFS in South Africa.

This study employed the Donabedian framework to assess the application of quality indicators in the implementation of the program. Within Donabedian’s structure-process-outcome conceptual framework, “structure” refers to the inputs, “process” denotes the quality of service delivery, and “outcome” represents the results achieved [32].

This study aimed to explore the HCPs’ experiences implementing the AYFS within Public Health Facilities in eThekwini, KwaZulu-Natal.

## 2. Materials and Methods

### 2.1. Study Setting

The research was conducted in four community health centers and three primary healthcare clinics located throughout the eThekwini health district in KwaZulu-Natal. The selected public health facilities all offered the comprehensive package of AYFS. The community health centers and primary healthcare clinics are strategically distributed across the northern, western, and southern regions of the eThekwini health district.

### 2.2. Research Design

An exploratory, descriptive, and contextual design was employed to explore the HCPs’ experiences with the implementation of the AYFS. This study utilized a qualitative approach to extract rich, in-depth, quality data. The detailed data facilitated an in-depth exploration and description of HCPs’ experiences in delivering AYFS. The research objective was met through direct engagement with individual participants through a semi-structured interview guide. This approach enabled the researchers to gain a deeper understanding of the HCPs’ experiences in their professional environments.

### 2.3. Research Participants

The target population for this study comprised HCPs working in public health facilities that deliver AYFS. The participants were deemed appropriate because they were the ones implementing the services. The inclusion criteria specified that eligible HCPs must provide AYFS and express willingness to participate in interviews and audio recordings. Conversely, the exclusion criteria encompassed individuals with less than one year of experience in AYFS and those on leave during the data collection period.

### 2.4. Sampling and Recruitment

A purposive sampling technique was used to sample HCPs who met the inclusion criteria. This enabled the researcher to select suitable, purposeful, information-rich samples to help clarify research questions and to obtain their perspectives on implementing AYFS [33,34,35]. A sample of eight HCPs from different facilities providing AYFS was approached for participation in the study. The participants were chosen because they were knowledgeable about the AYFS; thus, a small sample size was appropriate for this study.

### 2.5. Data Collection

Data were collected after receiving ethical clearance from gatekeepers. Data were gathered between August and December of 2023. Each potential participant received an information package that included an information letter about the study, a declaration, and an informed consent form. The researcher provided background information regarding the study, emphasizing that participation was voluntary and ensuring confidentiality of the data. Participants were informed that the interview would be recorded, and upon their decision to participate, they were asked to sign and provide verbal consent for the audio recording.

Data collection was conducted through in-depth individual interviews using a guide adapted from the WHO Healthcare Provider interview tool [36]. The tool highlighted the structural and process characteristics of the AYFS. Structural characteristics refer to the accessibility, appropriateness, and effectiveness of the services, while process characteristics are related to acceptability and equity. The open-ended format encouraged participants to express their thoughts freely. Interviews were conducted face-to-face in English, a language understood by the HCPs, and were audio recorded. Data saturation occurred following the eighth interview, as no additional themes emerged despite further probing.

### 2.6. Data Analysis

Data were analyzed using Braun and Clarke’s six phases of reflexive thematic analysis (RTA) [37,38], as cited in [39]. Thematic analysis enables researchers to engage deeply with the data, allowing for reflection, clarification, and intuitive insights during the analytical process [37]. The researchers carefully reviewed each interview prior to and during verbatim data transmission to understand the main topics addressed in each session. The coding process commenced with the identification of key codes that could facilitate theme development. Themes were identified and analyzed based on commonalities in meaning. The overlapping themes were integrated. The main themes and their respective sub-themes were established. The themes were evaluated for their significance in analyzing the data concerning the research question. Findings were reported following the Standard for Reporting Qualitative Research guidelines [40].

### 2.7. Trustworthiness

To assess the trustworthiness of the qualitative design, this study utilized the criteria established by Lincoln and Guba [41], which include credibility, transferability, dependability, and confirmability. To ensure credibility, the interviews were recorded with the participants’ consent and transcribed verbatim to accurately reflect their opinions. Each interview lasted approximately 45 min. Member checking was employed to confirm that the researcher accurately understood the participants’ meanings. A comprehensive description was provided, allowing participants’ words to be represented authentically where appropriate.

Transferability was supported by delivering a detailed account of the study population, setting, sample, sample size, data collection techniques, number of data collection sessions, and the duration of the data collection and analysis process [33]. Additionally, the researcher provided rich descriptions of the data to enable others to determine whether the findings could be applicable to other research contexts. Dependability was achieved through a detailed outline of the data collection and processing methods. The raw data were compared with the study’s findings and interpretations to facilitate a research audit. Agreement on the emergent themes was reached between the researcher and the research supervisor. Confirmability was established by ensuring that the research findings accurately reflected the experiences and perspectives of the informants. The transcribed data were validated by reviewing the recordings to confirm the accuracy of the transcription. The researchers did not allow their beliefs and assumptions to influence the research process by verifying the themes, sub-themes, and quotes with the research team.

### 2.8. Ethical Considerations

Informed consent: Each possible volunteer received an information package regarding the research, including the risks and benefits. Participation was entirely optional, and individuals could resign from the study at any time without fear of repercussions. Those willing to participate in the study were made to sign the informed consent. Anonymity and confidentiality were preserved by using pseudonyms to prevent data from being connected to participants. The researcher ensured the participants that the research does not hurt them. All participants were treated with respect, and their thoughts, feelings, and experiences were valued.

## 3. Results

### 3.1. Socio-Demographic Characteristics of the Participants

Eight in-depth interviews were conducted with black South African HCPs, with only one participant being male. The ages of the HCPs ranged from 34 to 50 years. Seven participants were trained as professional nurses, while one was trained as an auxiliary nurse. The level of experience in AYFS among the HCPs ranged from 1 to 14 years, as presented in Table 1.

### 3.2. The Experiences of HCPs in Implementing the AYFS

This section outlines the findings regarding the experiences of HCPs in implementing the AYFS within the public health facilities in eThekwini. The study identified four primary themes: (1) appropriateness of services, (2) competency, (3) accessibility of AYFS, and (4) adherence to the principles of beneficence and non-maleficence. Additionally, eleven sub-themes were identified within these themes. A summary of each theme and representative sub-themes extracted from the data is provided below in Table 2.

### 3.3. Theme 1: Appropriateness of Services

Appropriateness of service refers to the availability of services that cater for the unique needs of adolescents and youth. The participants indicated that they offered services tailored to the unique needs of adolescents. This theme encompasses six sub-themes related to the services provided within the AYFS. These sub-themes include a comprehensive package of adolescent services, outreach programs for educational institutions, continuity of care, prioritization of patients’ needs, maintenance of adequate supplies, resources, and equipment, and the availability of communication channels.

#### 3.3.1. Sub-Theme 1.1: Comprehensive Package of Adolescent Services

A comprehensive package of adolescent services refers to a single-point model of care. All eight participants interviewed reported delivering all services to adolescents and youth accessing the AYFS. They indicated their provision of syndromic management of STIs, treatment of opportunistic HIV infection, contraception, emergency contraception, pregnancy counseling and testing, pre- and post-test counseling, antenatal and postnatal care, male medical circumcision (MMC), condom distribution, post-exposure prophylaxis (PEP), and pre-exposure prophylaxis (PrEP). This is illustrated in the following statements:

“*I offer syndromic management of STIs, treatment of opportunistic HIV infection, contraception, emergency contraception, pregnancy counseling and testing, pre- and post-test counseling, ante and postnatal care, MMC, which is male medical circumcision, condom distribution, PEP, and PrEP*”

(Participant 6)

One measure of the appropriateness of adolescent services is the availability of the essential services in the AYFS.

#### 3.3.2. Sub-Theme 1.2: Community Outreach Programs

Community outreach programs serve as a strategy to improve access to healthcare providers. Participants in this study detailed the activities and festivities they organized to promote the AYFS program within the community. They shared various initiatives aimed at raising awareness of the AYFS program offered at their facility with the following:

“*Health education is our power tool to cascade the message that we do have the services, and we also use the war room*”

(Participant 3)

“*We do provide outreach programs to the schools. We are given time to educate all the children in the assembly*”

(Participant 4)

“*We send out invites to the nearest schools and pamphlets to those who come to the clinic to read about it while waiting, so they know that this program caters to the youth*”

(Participant 7)

Outreach programs increase the probability that the services provided are appropriate by bringing information and services closer to hard-to-reach adolescents.

#### 3.3.3. Sub-Theme 1.3: Continuity of Care

Continuity of care signifies the enhancement of the referral system for services unavailable at the facility. Participants in this study indicated that they refer adolescents and youth to appropriate service providers for services not offered, thereby ensuring continuity of care. The findings revealed that participants were familiar with the referral pathway. However, it was also evident from the participants that there was inconsistency in the follow-up of adolescents. The following statements illustrate these findings:

“*In this facility, we do not offer all the services that meet the adolescent’s needs, so we refer patients who require those services. We have a referral book where we write all the cases we refer. We also give a follow-up date to check on the progress*.”

(Participant 2)

“*If we have a patient that requires services that we do not render on that day, we refer the patient to the hospital, and then we need to have a way of tracking if the patient reached their referral hospital*.”

(Participant 5)

Suitable services for adolescents are those that ensure continuity of care for services unavailable on-site.

#### 3.3.4. Sub-Theme 1.4: Prioritization of Patients’ Needs

Allocating adequate time for consultations with adolescents is crucial to encourage them to express their concerns and issues. This approach also allows adolescents the opportunity to ask questions, obtain relevant information, and make informed decisions. Participants in this study reported that they devoted sufficient time to patient care and prioritized the needs of their patients. This is exemplified by the following statement:

“*I keep the door closed, not even closed but locked, and then my cell phone is silent*”

(Participant 3)

“*When I am with clients, I tell them I have all the time to listen to them to help solve their issues*.”

(Participant 7)

HCPs who demonstrate effective communication and listening skills with adolescents can facilitate their disclosure of their unique requirements, which makes them feel that the service is appropriate for their needs.

#### 3.3.5. Sub-Theme 1.5: Maintenance of Adequate Supply, Resources, and Equipment

The availability of supplies, resources, and equipment promotes the acceptability of the services offered. All the participants in this study confirmed that they ensured the AYFS were equipped with the necessary equipment, supplies, and essential services to deliver effective adolescent care. The following statement demonstrates this:

“*Order our supplies in advance. Ensure that I have everything that adolescents require. If a machine breaks down, we send it for repairs. We usually have spare machines*”

(Participant 1)

Despite participants mentioning ordering the supplies in advance, shortages still occurred, hindering services provision for adolescents. The availability of supplies, resources, and equipment enhances the utilization of services, as adolescents regard them as appropriate.

#### 3.3.6. Sub-Theme 1.6: Availability of Communication Channels

Effective communication channels are crucial within the AYFS to ensure participants feel engaged in its operations. The participants in this study indicated that suggestion boxes were available in most AYFS, offering adolescents a platform to communicate, whether to express compliments, raise concerns, or make suggestions. The following statement supports this assertion:

“*The facility has a suggestion box for patients to submit suggestions, complaints, or compliments*”

(Participant 2)

Although suggestion boxes are present in the facilities, the provision of feedback remains uncertain. Adolescent participation in planning and monitoring enhances confidence among both healthcare providers and adolescents regarding the appropriateness of service delivery in terms of location, timing, and methodology.

### 3.4. Theme 2: Healthcare Provider Competency

The participants indicated that they have achieved a high level of competency in facilitating the implementation of AYFS. The sub-theme that informed this theme was acquiring appropriate training.

#### 3.4.1. Sub-Theme 2.1: Acquiring Appropriate Training

As an HCP is working with adolescents and youth, it is essential to undergo training or in-service education. The training in AYFS equips the HCPs with the necessary competencies to effectively deliver the required service package to this population. The participants in this study reported having received training to address adolescent-related concerns. The following statements provide confirmation of this:

“*So, I’m a trained AYF champion, I’m also trained in family planning, HIV testing and counseling, and I have mental health qualifications*.”

(Participant 4)

“*We were given training in dealing with different groups, meaning we got sensitization on the key population to serve them. We were also given training in dealing with teenagers. I went for training for AYFS, so there we were learning about many things, including gender-based violence, dealing with teenagers, key populations, and all those things*”

(Participant 5)

### 3.5. Theme 3: Accessibility of AYFS

Promoting the accessibility of the AYFS is essential to enhancing the well-being of adolescents. In this study, the participants provided insights into the accessibility of AYFS for the target population, specifically regarding the distance traveled to these services. Three sub-themes contributed to this overarching theme.

#### 3.5.1. Sub-Theme 3.1: Geographic Location

The facility’s location, distance traveled, and public transport availability influence the accessibility of the AYFS. The participants indicated that the AYFS is easily accessible to adolescents, as it is within walking distance of their residence. Additionally, it was noted that even when adolescents need to utilize transportation, the costs are reasonable given its proximity to their homes. This finding was corroborated by participants who stated:

“*I would say it is easily accessible because most of the time, we are getting students, and the residents are very close to the clinic, so it’s within walking distance*”

(Participant 8)

“*It is easily accessible because there is a rank in the hospital where they are dropped off at the gate, and even if they are using a taxi, it’s less than 10 minutes. Most of the schools I mentioned around us are 10 minutes apart because sometimes they even start here before they go to school, which shows that it’s accessible and time is limited*”

(Participant 4)

#### 3.5.2. Sub-Theme 3.2: Appropriate Waiting Turnaround Period

The waiting period experienced by individuals in the AYFS can serve as either a deterrent or an enabler in accessing services. Participants reported the duration adolescents wait to be seen by HCPs, as illustrated in the following statements.

“*We have a tool we use, and the waiting time can be three to four hours*”

(Participant 4)

“*For me, in particular, because I am focusing on this age group, it does not take long. I do not think they take more than an hour in the clinic*”

(Participant 2)

“*The youth are prioritized. They do not join the queue. We have a policy that they need to be served if they come*”

(Participant 3)

The results show variation in the waiting times, likely due to high patient volume, staff shortages, and an inadequate triage system.

#### 3.5.3. Sub-Theme 3.3: Visible Signage to Provide Accessibility to the AYFS

The display of signage promoting AYFS in the facility is essential for encouraging service utilization. Adolescents exhibit apprehension in enquiring about sources of assistance. In this study, some participants reported that their facilities have signage that informs and directs adolescents regarding the services available to them. Other participants noted that their facilities lack signage to inform and guide adolescents regarding the services available and their locations. This is demonstrated in the following statements:

“*We have signs at the triage displayed on the notice board and then in the passages, like the ones that even have the arrows that show this area, even outside, that I’m working in*”

(Participants 1, 4)

“*Currently, we do not have signage directing them to this area*”

(Participants 2, 3, 5, 6, 7)

“*We do not have a sign stating that we offer AYFS in the facility. We only have A4 posters that state the youth-zone*”

(Participant 8)

Availability of signage promotes ease of access to the AYFS.

### 3.6. Theme 4: Adherence to the Principles of Beneficence and Non-Maleficence

HCPs must consistently adhere to the principles of beneficence and non-maleficence in their care delivery. The participants emphasized their ongoing commitment to maintaining privacy and confidentiality when caring for adolescents. They reported demonstrating respect towards adolescents to foster an environment in which these individuals feel comfortable discussing their concerns. This theme is supported by two sub-themes.

#### 3.6.1. Sub-Theme 4.1: Maintaining Confidentiality and Privacy

Maintaining confidentiality is essential when interacting with adolescents, as insensitivity to their concerns may hinder access to the AYFS. Participants indicated their commitment to upholding confidentiality and anonymity, as demonstrated by the following statements:

“*We offer them privacy. We close the door. OK, I tell them whatever you discuss stays within the team or between whoever we’re conversing with. Unless it’s something life-threatening like suicidal cases, but otherwise, we don’t discuss anything with anybody*”

(Participant 5)

“*All the healthcare workers are trained on the youth AYFS policy, so they know the importance of maintaining confidentiality*”

(Participant 4)

#### 3.6.2. Sub-Theme 4.2: Preservation of Human Dignity

Adolescents value respect and sensitivity from HCPs to be able to share the underlying problems that may be the reason for not visiting the facility. The participants indicated that they foster an environment in which adolescents feel welcomed and respected, thereby ensuring that they are comfortable expressing their concerns. The following statements support this assertion:

“*They are made to feel welcome. Regarding comfort, we ensure our youth are treated as adults. They are treated with respect. They need to be treated in a consulting room where there is a closed door, so whenever they are talking to you, nobody will hear anything from the outside, just because the door is open*”

(Participant 4)

“*So, I try as much as I can to be at their level and ask them in a friendly manner and try to show that I understand and that they can tell me anything and I won’t be judgmental; I’ll only help them so it’s easier for them to talk to me*”

(Participant 6)

## 4. Discussion

The study aimed to explore the HCPs’ experiences in implementing the AYFS in public health facilities in eThekwini, KwaZulu-Natal. The findings indicated that HCPs predominantly expressed satisfaction with their involvement in implementing AYFS, despite some perceived constraints. The implementation improved with appropriate services, ease of access to facilities, and provider competence. However, adolescents faced longer waiting times for consultations, which hindered service utilization. There were inconsistencies in follow-ups for individuals referred to other services, risking loss of care.

Participants reported that the AYFS provided a comprehensive package of services tailored to meet the needs and concerns of adolescents. These findings align with those of a scoping review conducted in low- and middle-income countries, which highlighted the importance of an appropriate package of services, facility characteristics, providers’ competencies, adolescent health literacy, community support, and adolescent participation [42]. The results also correspond with the findings of southern Ethiopia and Ghana. The availability of essential AYFS packages, HIV and STI treatment algorithms, contraceptives, supplies and equipment, a working referral and feedback system, and qualified healthcare professionals were all factors that improved the implementation of AYFS in Ethiopia [43,44]. Adolescent SRH education materials, standard guidelines, protocols, and policies, trained healthcare professionals, and the National Health Insurance Scheme (NHIS) covering contraceptive and family planning (FP) services for adolescents made implementation easier in Ghana [45,46]. In contrast, findings from Rwanda indicated that certain services were unavailable due to the established SRH healthcare package for health facilities [47]. The disparity between the Rwandan study and those from other LMICs may be attributed to the recent scaling up of healthcare programs in Rwanda, which has resulted in a separation of SRH services from AYFS.

The study findings indicated that the HCPS implemented outreach programs that fostered community engagement with educational institutions and improved adolescent health literacy through public announcements and brochure distribution. These findings align with the existing literature, which suggests that educational messages and community initiatives effectively enhance adolescents’ and community members’ knowledge and understanding of SRH services, thereby increasing service uptake [48,49]. Furthermore, these findings are consistent with those from a systematic review demonstrating that AYFS were promoted through local radio stations, posters, magazines, sporting events, and entertainment [50]. In contrast, studies from Guinea and Ethiopia indicated that the absence of demand-creation initiatives in schools and communities led to a knowledge deficit, thereby obstructing adolescents’ and youth’s access to SRH services [51,52]. Improving awareness and understanding of AYFS and the services provided significantly influenced the utilization of these services among adolescents [53].

The HCPs indicated that they maintained continuity of care by prioritizing the needs of adolescents, referring those requiring services not available on-site to appropriate facilities. However, there was no standardized method for tracking these referrals. These findings align with the research conducted in Rajasthan, India, where HCPs referred adolescents with substance abuse, alcohol addiction, STIs, menstrual pain, unwanted pregnancy, and nocturnal emissions to more qualified healthcare providers [54]. Conversely, these findings contrast with those from the United States, where HCPs reported that obtaining referrals and accessing speciality services was challenging due to a complex appointment process and extensive paperwork [55]. Additionally, HCPs expressed their commitment to regularly assess and identify deficiencies in supply. This observation is consistent with findings from north-west Ethiopia, where factors such as the availability of competent providers, medications, and supplies facilitated the implementation of AYFS [56]. The similarity in these findings may be attributed to a shared emphasis on the importance of continuity of care within healthcare systems.

Healthcare providers involved in this study demonstrated competence, having received training on delivering AYFS. According to the study findings, Malaysian HCPs provided care in a friendly manner, actively listened to adolescents’ concerns, offered quality counseling, and were prepared to deliver the necessary information [57]. These findings contrast with those from northern Ethiopia, where the structural quality dimension was compromised by a lack of adequately trained health service providers, insufficient engagement of adolescents and youth in facility governance, and the absence of guidelines, protocols, and procedures [58].

Participants in this study indicated that the AYFS location was accessible, with most youths able to walk to it, while others could utilize public transportation. These findings align with those from Rwanda, where adolescents traveled for less than 30 min to reach the AYFS [47]. Additionally, other literature supports this observation, noting that the location of the AYFS and its proximity to public transport were viewed as convenient [49,59]. Research has shown that the location of health facilities is significantly associated with adolescents’ utilization of SRH services [60]. To enhance visibility, it is recommended that service information, such as a signpost, be provided for easy identification [43]. The consistency of these findings may be attributed to accessibility being a key factor in effective AYFS, as highlighted by the WHO.

The findings of this study indicate that HCPs adhered to the principles of beneficence and non-maleficence by upholding privacy, confidentiality, and demonstrating respect towards adolescents. HCPs reported that they consistently reassured adolescents of the confidentiality of matters discussed during consultations. Adolescents highly value respect and a non-judgmental attitude from HCPs, as this fosters an environment where they feel comfortable communicating their issues with individuals who understand and respect them. Privacy and confidentiality are critical factors influencing the utilization of services. These findings are consistent with the existing literature from studies conducted in Ghana and Rwanda, which noted that health facilities provided private rooms for consultations to ensure privacy [45,47,49]. Additionally, these results align with a Nigerian study that emphasized the creation of a safe space for adolescents by HCPs [61]. Further literature from New Zealand, Ghana, and Malawi reflects similar sentiments, with HCPs demonstrating respect, non-judgment, friendliness, and a commitment to thoroughly addressing individuals’ concerns [45,49,59]. Establishing a trustworthy relationship with HCPs was identified as essential for New Zealand adolescents, enabling them to communicate openly, due to their concerns about being judged based on their behavior or health literacy [59]. These adolescents appreciated HCPs who consistently assured them of the confidentiality of their discussions. If implemented effectively, the future of AYFS will be characterized by youth-driven, digitally enabled, integrated, and holistic care, contributing to universal health coverage. The implications of these findings are to enable practice improvements that will ensure thorough implementation by ensuring consistent follow-up and enhancing utilization by shortening the waiting times, and adequate signage for ease of access to AYFS in the country. Policy can be enacted to ensure digital referral, tracking, and follow-up of cases to enable efficient care of youths in KwaZulu-Natal.

## 5. Conclusions

The study indicated that HCPs in the public health facilities in eThekwini generally reported positive experiences in the implementation of AYFS. The findings demonstrated that AYFS was delivered as a comprehensive package addressing the needs of adolescents, with HCPs actively engaging in community outreach, ensuring service accessibility, promoting health literacy, and facilitating continuity of care. Providers demonstrated competence and adhered to essential ethical principles, including confidentiality, respect, and non-judgmental care, which are critical for enhancing adolescent trust and service uptake. These findings align with the existing literature from various low- and middle-income countries, emphasizing the significance of provider competency, community engagement, and adolescent-centered care in the implementation of AYFS.

### 5.1. Limitations

The study concentrated solely on the experiences of HCPs within a single district, which limits its generalizability of the findings across broader geographic or health system contexts. Additionally, the research did not incorporate the perspectives of adolescents, which would have contributed to a more comprehensive understanding of the effectiveness and acceptability of AYFS. Furthermore, the reliance on qualitative self-reporting may have introduced social desirability bias, potentially affecting how HCPs articulated their practices and experiences.

### 5.2. Recommendations

There is a pressing need to enhance HCPs’ educational programs, particularly in areas such as continuous professional development in adolescent health, communication, and ethical practice. Prioritizing these initiatives is essential to maintaining provider competency. Community outreach activities can be enhanced by expanding demand-creation initiatives within schools and communities to raise awareness and promote service utilization among this vulnerable population.

The study findings indicated that HCPs had a positive experience in implementing AYFS; however, they reported inconsistencies in the referral system due to a lack of tracking mechanisms. Therefore, it is imperative to improve the referral system by streamlining processes and implementing tracking methods to ensure timely and effective continuity of care for services not available on-site. This can be achieved through the implementation of an electronic referral platform that enables healthcare professionals (HCPs) to send, receive, and track referrals in real time. In South Africa, a centralized information management system could be established to consolidate patient data across various facilities. Additionally, adolescent referrals and follow-ups can be facilitated through mobile applications.

Additionally, increasing the visibility of AYFS within healthcare facilities is crucial. This can be achieved by providing clear signage and informational materials to help adolescents easily identify and access AYFS locations. Further mixed-method studies should be conducted to capture the perspectives of adolescents and youths, facilitating a more balanced understanding of AYFS.

## Figures and Tables

**Table 1:** The socio-demographic characteristics of the participants.

Pseudonyms	Gender	Age Bracket	Qualification	Experience in AYFS
Participant 1	Female	45–50	Professional nurse	1 year
Participant 2	Female	41–45	Professional nurse	6 years
Participant 3	Female	30–40	Professional nurse	10 years
Participant 4	Female	41–45	Professional nurse	4 years
Participant 5	Female	30–40	Professional nurse	1 year
Participant 6	Female	30–40	Auxiliary nurse	1 year
Participant 7	Male	30–40	Professional nurse	8 years
Participant 8	Female	30–40	Professional nurse	6 years

**Table 2:** Summary of themes and sub-themes.

Themes	Sub-Theme	Quotes	Meaning
Appropriateness of services.	Provide a comprehensive adolescent services package.	We offer HIV tests, ART to HIV-positive clients, PREP, PEP, family planning, and both female and male condoms. Health Education and Antenatal Care. Specializing more in prevention. For the termination of pregnancy, we refer them to the secondary level. Health education regarding diets, counseling them, and offering screening services for non-communicable diseases. Youth-Zone.	Participants revealed that they offered most services addressing adolescents’ concerns; when services are not applicable, a referral is made.
	Outreach programs for educational institutions.	Use the war room in Chesterville. Engage in youth activities and celebrations involving the community. We engage the community and invite them to empower themselves to gain knowledge.	
	Continuity of care.	We do not offer all the services. We refer patients. Have a referral book. Follow-up date to check on the progress. Refer the patient back to us once they finish their part.	
	Prioritization of patients’ needs.	Youth is prioritized. Don’t join the queue. We have a policy.	
	Maintenance of adequate supply, resources, and equipment.	Order our supplies in advance. Ensure that I have everything that adolescents require. We sent a broken machine for repairs. Usually have spare machines.	
	Availability of communication channels.	A suggestion box where they can put their suggestions and complaints.	
Healthcare provider competency.	Acquired appropriate training.	I’m a trained AYF champion, having received an in-service education. I am trained in family planning, HIV testing, and counseling, and I have mental health qualifications. Trained in stress management activities. How to deal with rape cases, termination of pregnancy.	Participants reported having received appropriate training in AYFS.
Accessibility of AYFS.	Geographic location.	It is easily accessible, we are getting students, and the residents are very close to the clinic. It’s within walking distance.	Ease of access was reported as facilities are within the community.
	Waiting period turnaround.	We have a tool we use. Waiting time can be three to four hours.	
	Visible signage to provide accessibility to the AYFS.	We have signs at the triage displayed on the notice board and then in the passages, like the ones that even have the arrows that show this area. Currently, we do not have signage directing them to this area.	
Adherence to the principles of beneficence and non-maleficence.	Maintaining confidentiality and privacy.	It’s just me and the client in the room. No one interrupts us. Also, reassured the patient. Everything we discussed would be between them and me. No other person would know it.	The HCPS stated that they maintain privacy and confidentiality and use a non-judgmental approach.
	Preservation of human dignity.	They are made to feel welcome. Ensure our youth are treated as adults. Treated with respect.	

## Data Availability

Data from this study are the property of the University of KwaZulu-Natal and may be made available upon request from the university or the study’s authors.

## Abbreviations

The following abbreviations are used in this manuscript:

AYFS	Adolescent and Youth-Friendly Services
HCPs	healthcare providers
HIV	human immunodeficiency virus
LMICs	lower-middle-income countries
SRH	sexual and reproductive health
SRHR	sexual and reproductive health and rights
STIs	sexually transmitted infections
WHO	World Health Organization
